# Antibiotic Use during the COVID-19 Pandemic in a Tertiary Hospital with an Ongoing Antibiotic Stewardship Program

**DOI:** 10.3390/antibiotics10091056

**Published:** 2021-08-31

**Authors:** Oryan Henig, Orli Kehat, Suzy E. Meijer, Amanda Chikly, Ahuva Weiss-Meilik, Eyal Egoz, Ronen Ben-Ami, Yael Paran

**Affiliations:** 1 Infectious Disease and Epidemiology Department, Tel Aviv Sourasky Medical Center and Sackler School of Medicine, Tel Aviv University, Tel Aviv 6423906, Israel; suzymeijer@gmail.com (S.E.M.); amandac@tlvmc.gov.il (A.C.); Ronenba@tlvmc.gov.il (R.B.-A.); yaelp@tlvmc.gov.il (Y.P.); 2 Medata AI Center, Tel-Aviv Sourasky Medical Center, Tel Aviv 6423906, Israel; orlyk@tlvmc.gov.il (O.K.); ahuvawm@tlvmc.gov.il (A.W.-M.); eyaleg@tlvmc.gov.il (E.E.)

**Keywords:** antibiotic stewardship, COVID-19, DOT (days of treatment)

## Abstract

During the recent pandemic, the fact that the clinical manifestation of COVID-19 may be indistinguishable from bacterial infection, as well as concerns of bacterial co-infection, have been associated with an increased use of antibiotics. The objective of this study was to assess the effect of targeted antibiotic stewardship programs (ASP) on the use of antibiotics in designated COVID-19 departments and to compare it to the antibiotic use in the equivalent departments in the same periods of 2018 and 2019. Antibiotic consumption was assessed as days of treatment (DOT) per 1000 patient days (PDs). The COVID-19 pandemic was divided into three periods (waves) according to the pandemic dynamics. The proportion of patients who received at least one antibiotic was significantly lower in COVID-19 departments compared to equivalent departments in 2018 and 2019 (Wave 2: 30.2% vs. 45.6% and 44.9%, respectively; Wave 3: 30.5% vs. 47.8% and 50.1%, respectively, *p* < 0.001). The DOT/1000PDs in every COVID-19 wave was lower than during similar periods in 2018 and 2019 (179-282 DOT/1000PDs vs. 452-470 DOT/1000PDs vs. 426-479 DOT/1000PDs, respectively). Moreover, antibiotic consumption decreased over time during the pandemic. In conclusion, a strong ASP is effective in restricting antibiotic consumption, particularly for COVID-19 which is a viral disease that may mimic bacterial sepsis but has a low rate of concurrent bacterial infection.

## 1. Background

SARS-CoV-2 emerged in December 2019 and has since imposed an enormous burden on healthcare systems worldwide. The clinical manifestations of severe COVID-19 may be indistinguishable from those of bacterial infection, characterized by acute onset, fever, increased inflammatory markers, and organ failure [1]. This clinical uncertainty as well as the lack of effective treatment options for SARS-CoV-2 were among the reasons for the widespread use of antibiotics early in the pandemic [2]. Most of the efforts in healthcare and research were targeted towards finding an effective treatment for COVID-19, while restricting the use of antibiotics was not a top priority [3]. 

Several studies and meta-analyses have shown that around 70% of hospitalized patients with COVID-19 were treated with antibiotics during hospitalization, mainly due to concerns of bacterial co-infection [2,4]. However, detailed review of patient data shows that only around 6% of hospitalized COVID-19 patients have microbiologically confirmed bacterial co-infection [5,6]. This is in contrast to influenza, where bacterial co-infection was reported in around 30% of hospitalized patients [7]. The antibiotics most commonly used were agents targeting community-acquired pneumonia, including tetracyclines, macrolides, and cephalosporins [2]. A recent meta-analysis of seven randomized clinical trials that evaluated the impact of azithromycin on clinical outcomes of COVID-19 patients has shown no favorable effect on mortality or length of stay [8].

At the Tel Aviv Medical Center, an antibiotic stewardship program (ASP) was implemented early in the course of the COVID-19 pandemic. The intervention was formatted differently than the usual ASP conducted before the pandemic and was based on the principles described below.

The objective of this study was to assess the effect of targeted antibiotic stewardship on antibiotic use in COVID-19 units. In our hospital, regular medicine departments were transformed into designated COVID-19 departments using the same medical staff. This study also looked to compare the antibiotic consumption in those designated COVID-19 departments to the antibiotic consumption in those same departments at the equivalent calendar months of 2018 and 2019 (i.e., equivalent departments), before the COVID-19 pandemic.

## 2. Methods

### 2.1. Study Population and Setting

This was a retrospective observational study performed at a 1400-bed tertiary hospital with 9 medical wards. The cohort included adult inpatients who were hospitalized between March 2018 and April 2021. COVID-19 patients were defined as patients who were diagnosed by RT-PCR assay. Designated COVID-19 departments were placed in specially adjusted wards where patients were admitted under strict airborne and contact precautions. During the study period, 3 surges of the COVID-19 pandemic (COVID-19 waves) were identified: period 1 (wave 1) between March and mid-April 2020; period 2 (wave 2) between June and November 2020, and period 3 (wave 3) between December 2020 and April 2021.

Ethics approval for this study was obtained from the institutional review board along with a waiver of written informed consent (Approval #TLV-020620).

We compared antibiotic use in medical departments during each period with antibiotic use in the equivalent departments in the years 2018 and 2019 during the same calendar months. Medical departments that were appointed for the cohorting of CPE or VRE carriers were excluded from the analysis because they contained a different type of patient cohort and different treatment protocols. 

### 2.2. The Institution’s Antibiotic Stewardship Program

Our institution’s regular ASP includes providing our physicians with local guidelines on antibiotic treatment of several infectious diseases as well as conducting weekly meetings with many departments of different specialties that concentrate on optimizing antimicrobial treatment. 

During the pandemic, an adjusted, more intensive ASP was put in place for the designated COVID-19 departments: Local COVID-19 treatment guidelines were formatted by a team consisting of an infectious disease specialist, an intensive care physician and an internal medicine physician. These guidelines were updated every 1–2 weeks.

A special introduction briefing was held before a department turned into a designated COVID-19 department. During this meeting, an infectious disease consultant discussed the most updated version of the institution’s COVID-19 treatment guidelines, presented the latest research publications on the treatment of COVID-19 as well as the local experience that was accumulated so far.

Daily rounds with the infectious disease specialist and the department’s medical team were conducted on every weekday.

Every patient admitted to the COVID-19 department was presented by the medical team to the infection disease consultant. During daily rounds, a revision of microbiology results was performed, as well as a clinical evaluation. Treatment was adjusted accordingly.

This consultation was performed by one specific infectious disease consultant assigned to the department in order to maximize continuity and collaboration with the medical team.

### 2.3. Definitions and Data Collection

Data pertaining to demographics (age, gender), Charlson’s comorbidity index (CCI) without the age component, need for mechanical ventilation and dates of admission and discharge or death were extracted from the electronic health record (EHR). Antibiotic agents that were administered orally or parenterally were captured. Antibiotic consumption was measured in days of treatment (DOT) per 1000 patient days (PDs) according to the IDSA antibiotic stewardship guidelines [8]. One DOT represents the use of a single antibiotic on a given day. In addition to DOT, the date of first antibiotic administered as well as the indications for antibiotic treatment were extracted. 

### 2.4. Analysis

Descriptive statistics of the variables were used to characterize the cohort: categorical variables were presented as percentages and continuous variables as medians and interquartile range (IQR). Categorical and continuous variables were compared using Chi squared tests and a Kruskal–Wallis tests, respectively. *p* < 0.005 was considered to be statistically significant and all *p*-values were two-sided.

DOT/1000 PDs of COVID-19 departments during each wave were calculated and presented with DOT/1000 PDs of the original equivalent departments during the same calendar months of 2018 and 2019.

## 3. Results

During the COVID-19 pandemic, a total of 1836 patients were hospitalized in COVID-19 departments with a mean age of 69 years. Fifty eight percent were male. 

As presented in Table 1, patients who were admitted to COVID-19 departments were younger and had fewer comorbidities than non-COVID patients during the same calendar months of 2018 and 2019. However, the proportion of patients who were mechanically ventilated and the length of hospital stay were higher for COVID-19 patients versus non-COVID patients. Specifically, 18.2% of hospitalized patients during the first wave of 2020 were mechanically ventilated compared to 6.2% and 5.4% of hospitalized patients in the equivalent departments in 2019 and 2018, respectively, *p* < 0.001; median LOS during the first wave of 2020 was 7.5 days (IQR 3.1–15.5 days) compared to a median LOS of 3.8 days (IQR 2–7.9 days) and 3.7 days (IQR 2.1–7.9 days) in 2019 and 2018, respectively.

### 3.1. Antibiotic Treatment during the COVID-19 Pandemic Compared to 2018 and 2019

Overall, among patients who were hospitalized in COVID-19 departments, 30.9% received at least one dose of antibiotic. The median time from hospitalization to start of the antibiotic treatment was 27 h (IQR 14.6–124.6 h), and the median duration of treatment was 5 days (IQR 2–8 days).

The proportion of patients who received antibiotics was significantly lower during the second and third wave of the COVID-19 pandemic compared to the proportion of patients who received antibiotics in the equivalent departments during the same calendar months of 2018 and 2019 (Wave 2: 30.2% vs. 45.6% and 44.9% in 2018 and 2019, respectively. Wave 3: 30.5% vs. 47.8% and 50.1% in 2018 and 2019, respectively, *p* < 0.001 for both comparisons). During the first wave, a lower proportion of patients who were hospitalized in COVID-19 departments received antibiotics compared to patients who were hospitalized in the equivalent departments in 2018 and 2019, but this difference was not statistically significant (i.e., Wave 1: 37.8% of patients received antibiotics compared to 46.5% and 47.7% in 2018 and 2019, respectively). 

For all time periods, patients who were treated with antibiotics were older, had more comorbidities, and were more severely ill (higher proportion of mechanical ventilation, longer LOS and higher mortality rates) (Table 2).

**Figure 1:**
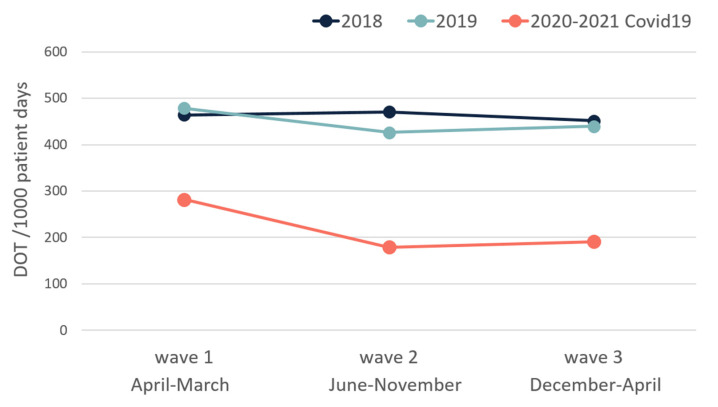
Cumulative DOT per 1000 patient days in each wave and in the same medical departments at the same calendar months of 2018 and 2019.

**Figure 2:**
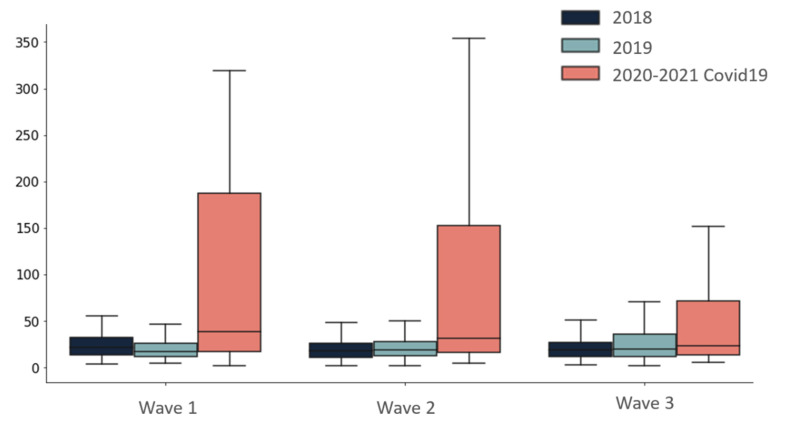
Time to first administration of antibiotics (in hours). *p* < 0.001 for the comparison of time to first administration of antibiotics in COVID-19 departments compared to equivalent departments in the same calendar months of 2018 and 2019.

The DOT/1000 PDs during each COVID-19 wave were lower than the DOT/1000 PDs in non-COVID-19 departments during the same calendar months (Figure 1). In addition, the time to beginning of antibiotic treatment was longer among patients who were hospitalized in COVID-19 departments compared to patients who were hospitalized in the equivalent departments in 2018 and 2019 (*p* < 0.001) (Figure 2). 

**Figure 3:**
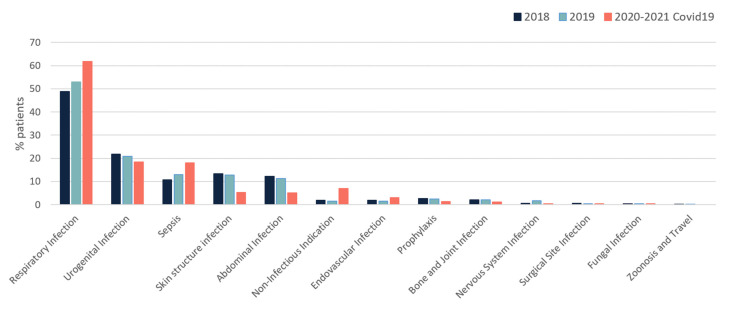
Indications for antibiotic treatment over 3 years, including 2020.

The most commonly used antibiotics in COVID-19 departments were second and third generation cephalosporins (52.2%), followed by anti-pseudomonal agents (28%), vancomycin (16%), and aminoglycosides (11%). The most commonly documented indications for antibiotic treatment were respiratory tract infection (62%), urinary tract infection (18%) and sepsis (18%) (Figure 3).

### 3.2. Antibiotic Treatment during the COVID-19 Pandemic. Comparison between the Different Waves

**Figure 4:**
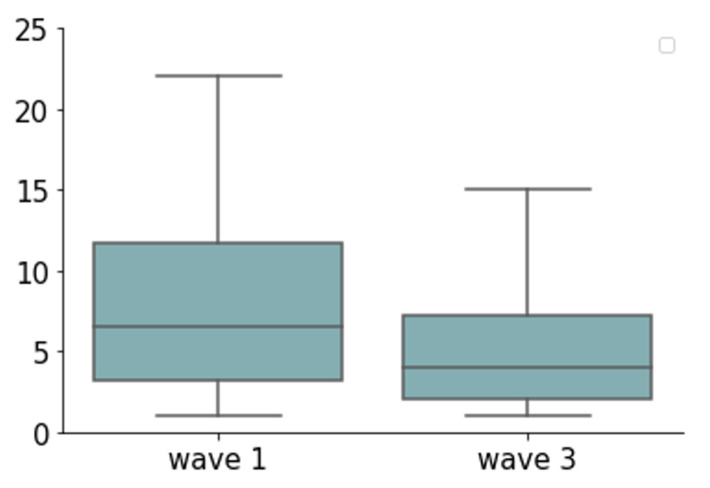
DOT of COVID-19 patients in the third wave compared to the first wave. DOT, days of treatment; *p* = 0.003.

During the COVID-19 pandemic, there was a decrease in the proportion of patients who received antibiotics in the third wave compared to the first wave (37.8% in the first wave vs. 30.5% in the third wave, *p* = 0.1), and the median DOT in the third COVID-19 wave was significantly lower than in the first wave (DOT first wave 6.5 days (IQR 3.25–11.75 days); median DOT third wave 4 days (IQR 2–7.25 days); median *p* = 0.003) (Figure 4). Of note, during the pandemic, the reduction in antibiotic use was not accompanied by increased mortality (23.8% in the first wave compared to 22.4% in the third wave, *p* = 0.39).

## 4. Discussion

In this retrospective study at a large tertiary hospital, the overall antibiotic consumption (both the proportion of patients treated with antibiotics and the DOT/1000 PDs) was lower among patients who were hospitalized in COVID-19 departments than that observed in the equivalent non-COVID-19 departments who were treated by the same medical teams during the same calendar months of 2018 and 2019. In addition, the rate of antibiotic consumption in our center during the pandemic (30.2–37.8%) was much lower than that described in most previous publications in the literature (70–95%) [2,9,10].

One of the observations that may have explained lower antibiotic use during the pandemic was that patients in the COVID-19 departments were younger than the non-COVID-19 patients and had fewer comorbidities. However, the proportion of COVID-19 patients with mechanical ventilation and length of stay were significantly higher among COVID-19 patients. These parameters are commonly associated with increased likelihood of nosocomial infections and potentially higher antibiotic use, which was not seen in our center, possibly owing to ASP interventions. 

In a report that was published early in the course of the pandemic, Abelenda-Alonso et al. reported a biphasic pattern of antibiotic use during the first pandemic wave between March and April 2020 [11,12]: the first phase was explained by empirical antibiotic therapy for suspected bacterial respiratory infection, and the second phase reflected the antibiotic treatment of patients admitted to intensive care units that were treated with broad-spectrum antibiotics for nosocomial infections. Indeed, a major proportion of bacterial infections in COVID-19 patients is due to nosocomial infections, including central-line-associated blood stream infections and ventilator-associated pneumonias that are typical for patients with prolonged hospitalization in intensive care settings [13]. 

Interestingly, the antibiotic consumption dropped dramatically between the first wave and the second and third waves, without changes in in-hospital mortality. We believe this represents a local learning curve and reflects the knowledge and experience that were accumulated globally throughout the pandemic. Similar findings were described by Parra J.C. et al., who reported a decrease in antibiotic use during the second wave compared to the first wave [14]. In the absence of therapeutic options, early in the pandemic the proportion of patients who received antibiotics reached 95% in a report from China [9,10] and over 70% in other geographic areas [2,15,16]. Data pertaining to the small proportion (6–8%) of patients with bacterial or fungal co-infections were published later in the pandemic [4,5,6,17]. 

Langford et al. found a similar proportion of patient treated with antibiotics in different healthcare settings (hospital and community) and across all age groups (pediatric and adults) [4]. More than 70% of physicians treating COVID-19 hospitalized patients were reported to prescribe antibiotics [18]. Moreover, the international WHO guidelines that were published at the beginning of the pandemic, in March 2020, recommended antibiotic treatment for COVID-19 patients who presented sepsis [19]. Later, when the clinical manifestations of SARS-CoV-2 infection became clear and it was realized that the clinical picture mimics bacterial infection while the incidence of bacterial co-infection is low, these guidelines were modified. The current WHO guidelines for COVID-19 [20] specifically mention not to prescribe antibiotics unless there is clinical suspicion of a bacterial infection.

We believe that the lower rates of antibiotic consumption in our hospital were the result of ongoing ASP activity starting at the opening of each COVID-19 department and continuing throughout the entire pandemic. The ASP meetings during the COVID-19 pandemic were formatted differently than the usual ASP meetings conducted before the pandemic in order to be able to customize to the rapid developments in knowledge and research. 

Similar reports from other areas support our findings. In a survey of antibiotic and antifungal prescribing from Scotland, 38% of hospitalized COVID-19 patients were treated with antibiotics. The authors attribute this low rate of antibiotic prescription to the “mature national stewardship program in Scotland and a coordinated national response to COVID-19” [11]. In a point prevalence survey from Singapore, only 6.2% of patients were on antibiotics at the time of the survey, but still the authors concluded that 40.5% of these were inappropriately prescribed. They also described that the antibiotics were more appropriately prescribed when reviewed by an infectious disease specialist than by non- infectious disease physicians [21]. This high intensity ASP approach is different from some of the published data where ASPs were suspended during the pandemic leading to higher antibiotic consumption [3,16]. 

Our study has several limitations. First, the retrospective design reduces control over multiple confounders. This is especially true regarding the comparison of COVID-19 patients to non-COVID-19 patients in the equivalent periods in the previous years due to the different patient populations. However, despite the differences in case mix between the two study populations of COVID-19- and non-COVID-19 departments, the indications for antibiotic treatment were similar, and in both populations, the most common indication was respiratory infection for which similar antibiotic prescription would be expected. Moreover, even though the case mix of the populations was different, the higher proportion of mechanical ventilated patients and the longer duration of hospitalization among COVID-19 patients would be expected to lead to higher antibiotic consumption. Second, we did not have any microbiological data and could therefore not present the proportion of bacterial co-infections. Although the most common indication for antibiotic treatment in COVID-19 departments was respiratory infection, the proportion of patients suspected of having a respiratory infection that actually had microbiologically documented infection is unknown. Lastly, the study is a single center study. 

## 5. Conclusions

In conclusion, this study shows a lower rate of antibiotic use in designated COVID-19 departments compared to similar departments in the years before. It also shows that antibiotic consumption decreased over time in association with a COVID-19-adjusted ASP. The rate of patients being treated with antibiotics compared to rates described in most published data was low. The importance of a high intensity ASP is especially true for COVID-19, which is a viral disease that presents an increase in inflammatory markers and multi-organ failure which mimics bacterial sepsis but has a low rate of concurrent bacterial infection. The importance of a well-established ASP is particularly relevant because of the massive impact of the pandemic on global health in a time where multidrug-resistant organisms are on the rise.

## Figures and Tables

**Table 1:** Medical COVID-19 departments (2020–2021) vs. equivalent wards in 2018 and 2019.

	Wave 1 (March–April)				Wave 2 (June–November)				Wave 3 (December–March)			
	2018	2019	2020-COVID	*p*-Value	2018	2019	2020-COVID	*p*-Value	2018	2019	2020-COVID	*p*-Value
N	482	484	143	-	2533	2521	933	-	1468	1855	760	-
age	73 (61,83)	75 (63,85)	69 (53,79)	*p* < 0.001	72 (60,84)	72 (59,83)	69 (55,81)	*p* < 0.001	74 (62,85)	73 (61,84)	69.5 (53,81)	*p* < 0.001
% males	50.8	50	59.4	NS	51.6	51.6	57.4	*p* < 0.01	52	51.9	57.8	*p* < 0.05
CCI	2 (0,3)	2 (0,3)	0 (0,2)	*p* < 0.001	1 (0,3)	1 (0,3)	1 (0,2)	*p* < 0.001	2 (0,3)	1 (0,3)	1 (0,3)	*p* < 0.001
LOS	3.7 (2.1,7.9)	3.8 (2,7.9)	7.5 (3.1,15.5)	*p* < 0.001	3.3 (1.9,7.1)	3.8 (2,7.8)	5.8 (2.4,13)	*p* < 0.001	4 (2.1,8.4)	4.3 (2.2,9.1)	5.1 (2.5,9.7)	*p* < 0.001
mechanical ventilation	5.4	6.2	18.2	*p* < 0.01	5.4	4.9	9.2	*p* < 0.001	7.6	5.6	9.1	*p* < 0.001
in-hospital mortality	12	14.3	23.8	*p* < 0.001	12	11.6	19.8	*p* < 0.001	13.3	13.2	22.4	*p* < 0.01
% given antibiotics	46.5	47.7	37.8	NS	45.6	44.9	30.2	*p* < 0.01	47.8	50.1	30.5	*p* < 0.001
DOT	6 (3,10.3)	6 (3,10)	6.5 (3.3,11.8)	NS	5 (3,9)	5 (3,9)	5 (3,8)	NS	5 (3,10)	5 (3,9)	4 (2,7.3)	*p* < 0.001
DOT/1000 patient days	464.1	478.7	282.1	-	470.8	426.2	179.4	-	451.7	439.7	191.1	-

LOS = length of stay, CCI = Charlson’s comorbidity index, DOT = Days of treatment. Age, DOT and LOS—medians and IQRs are shown.

**Table 2:** Comparison between treated and nontreated patients.

	2020			2019			2018		
	Antibiotic Treatment	No Antibiotic Treatment	*p*-Value	Antibiotic Treatment	No Antibiotic Treatment	*p*-Value	Antibiotic Treatment	No Antibiotic Treatment	*p*-Value
	N = 568	N = 1268		N = 2294	N = 2566		N = 2082	N = 2401	
Age [median, (IQR)]	74 (63,84)	66 (51,79)	*p* < 0.001	76 (64,86)	70 (57,81)	*p* < 0.001	76 (64,86)	71 (58,82)	*p* < 0.001
Gender (% male)	55.8	58.6		51.3	51.8		52.1	51.2	*p* = 0.29
Charlson’s comorbidity index [median, (IQR)]	2 (0,3)	1 (0,2)	*p* < 0.001	2 (0,3)	1 (0,3)	*p* < 0.001	2 (1,3)	1 (0,3)	*p* < 0.001
Length of stay [median days, (IQR)]	9.6 (5,19)	4.1 (2.1,8.9)	*p* < 0.001	5.8 (3.1,12.1)	2.8 (1.5,5.4)	*p* < 0.001	5.5 (3.1,10.8)	2.4 (1.4,5)	*p* < 0.001
Ventilation status (%)	19.7	5.4	*p* < 0.001	9.4	1.6	*p* < 0.001	11.1	1.8	*p* < 0.001
In-hospital mortality (%)	41.9	11.9	*p* < 0.001	20.5	5.3	*p* < 0.001	20.3	5.7	*p* < 0.001
DOT [median, (IQR)]	5 (2,8)	0 (0,0)	-	5 (3,9)	0 (0,0)	-	5 (3,9)	0 (0,0)	-

## Data Availability

The data presented in this study are available on request from the corresponding author.
